# Cytotaxonomy of *Eurypyga helias* (Gruiformes, Eurypygidae): First Karyotypic Description and Phylogenetic Proximity with Rynochetidae

**DOI:** 10.1371/journal.pone.0143982

**Published:** 2015-12-01

**Authors:** Ivanete de Oliveira Furo, Amanda Almeida Monte, Michelly da Silva dos Santos, Marcella Mergulhão Tagliarini, Patricia C. M. O´Brien, Malcolm A. Ferguson-Smith, Edivaldo H. C. de Oliveira

**Affiliations:** 1 Programa de Pós Graduação em Genética e Biologia Molecular, Universidade Federal do Pará, Campus Universitário do Guamá, Belém-PA-Brazil; 2 Laboratório de Cultura de Tecidos e Citogenética, SAMAM, Instituto Evandro Chagas, Ananindeua, PA, Brazil; 3 Universidade Federal do Pará, ICB, Faculdade de Biologia, Universidade Federal do Pará, Campus Universitário do Guamá, Belém-PA-Brazil; 4 Programa de Pós Graduação em Neurociências e Biologia Celular, Universidade Federal do Pará, Campus Universitário do Guamá, Belém-PA-Brazil; 5 Cambridge Resource Centre for Comparative Genomics, University of Cambridge Department of Veterinary Medicine, Cambridge, United Kingdom; 6 Instiuto de Ciências Exatas e Naturais, Universidade Federal do Pará, Belém-PA-Brazil; Universita degli Studi di Roma La Sapienza, ITALY

## Abstract

The sunbittern (*Eurypyga helias*) is a South American Gruiformes, the only member of Family Eurypigidae. In most phylogenetic proposals, it is placed in a more distant position than other families of the so-called “core Gruiformes”. Different studies based on molecular, morphological and biogeographical data suggest that the Eurypigidae is closely related to the kagu (*Rhynochetos jubatus*), the only species in Rynochetidae, another family not included in the core Gruiformes. Here, the karyotype of the sunbittern is described for the first time, by classical and molecular cytogenetics, using whole chromosome probes derived from *Gallus gallus* and *Leucopternis albicollis*. We found a diploid number of 80, with only one pair of biarmed autosomal macrochromosomes, similar to that observed in the kagu. Chromosome painting revealed that most syntenies found in the avian putative ancestral karyotype (PAK) were conserved in the sunbittern. However, PAK1, PAK2, and PAK5 corresponded to two chromosome pairs each. Probes derived from *L*. *albicollis* confirm that fissions in PAK1 and PAK2 were centric, whereas in PAK5 the fission is interstitial. In addition, there is fusion of segments homologous to PAK2q and PAK5. From a phylogenetic point of view, comparisons of our results with two other Gruiformes belonging to family Rallidae suggest that the PAK5q fission might be a synapomorphy for Gruiformes. Fissions in PAK1 and PAK2 are found only in Eurypigidae, and might also occur in Rynochetidae, in view of the similar chromosomal morphology between the sunbittern and the kagu. This suggests a close phylogenetic relationship between Eurypigidae and Rynochetidae, whose common ancestor was separated by the Gondwana vicariancy in South America and New Caledonia, respectively.

## Introduction

The diversification of Gruiformes, at least at the family level, seems to have happened almost entirely in the Upper Cretaceous [1]. Following the geographic isolation of New Caledonia, many species became endemic on this island. According to Ladiges & Cantrill [2], among the 106 bird species that occur in New Caledonia, 22% are endemic; similarly, 2432 (74%) of 3270 plant species are endemic. Nevertheless, cladistic biogeography indicates a strong evolutionary relationship between some endemic species on this island and those located in distinct geographic regions [2]. This relationship endorses the hypothesis of vicariance of Gondwana [3–4] that might also have involved the sunbittern (*Eurypyga helias*), from South America, and the kagu (*Rhynochetos jubatus*), from New Caledonia, both within the order Gruiformes.

The sunbittern, found in the tropical rainforests of Central and South America, is the only species of family Eurypygidae [5–7]. Several phylogenetic approaches have been applied to this species [8–11], however, the phylogenetic relationship between this species and other Gruiformes remains unclear [1, 10–11]. Nonetheless, the sunbittern is supposed to be closely related to *Aptonis*, extinct in New Zealand, and to the kagu (*Rhynochetos jubatus*) of New Caledonia [12]. In fact, ther close relationship between the sunbittern and the kagu is supported by studies based on DNA sequencing, indicating a common ancestry for Eurypigidae and Rhynochetidae [9, 13–14]. The consensus is that these two lineages were isolated geographically during the separation of the supercontinent Gondwana during the Jurassic, after the Pangea split [2, 12]. This geographic isolation, in addition to other selective pressures (e.g climate and environmental changes), caused the divergence of these species [12, 15].

Synapomorphies based on comparative cytogenetics can help elucidate evolutionary relationships of species with controversial phylogenies [16]. In birds, most phylogenetic studies using this approach have used whole-chromosome paints derived from *Gallus gallus* [17–20]. The study of chromosomal syntenies between chicken and two species of Gruiformes, *Fulica atra* and *Gallinula chloropus*, indicates conservation of macrochromosomes in both species. Two fusion events were identified: GGA4q and GGA5 and GGA6 and GGA7 [20]. Although only these two species of this order have been analyzed, the results were assumed to be characteristic of Gruiformes [20]. The use of different probe sets, such as those derived from the white hawk (*Leucopternis albicollis*), in which some of the macrochromosomes of chicken are divided into two or more pairs, would provide valuable confirmation of these rearrangements and the recurrence of breakpoints.

Hence, in order to investigate the phylogenetic position of the sunbittern, on which no cytogenetic data have been published previously, we investigated its karyotype by means of classical and molecular cytogenetics, applying whole chromosome paints derived from chicken (*Gallus gallus*) and white hawk (*Leucopternis albicollis*). The data obtained were used to infer the phylogenetic and biogeographic relationships of the sunbittern within Gruiformes.

## Materials and Methods

### Biological material and chromosome preparation

Experiments followed protocols approved by the Ethics Committee on the use of animals (CEUA-Universidade Federal do Pará, Permission Number: 070/2013). Mitotic chromosomes were obtained from fibroblast cultures using feather pulp, collected from three individuals (one male, two females) kept at Parque Mangal das Garças (Belém, PA, Brazil). We performed fibroblast culture following standard protocols, with modifications [21]: the tissue was dissociated mechanically and incubated in collagenase IV (0,5% in DMEM) for one hour (37°C), following addition of DMEM medium and centrifugation. After discarding supernatant, 5 ml of DMEM supplemented with fetal bovine serum (5%) was added, and this material was transferred to culture flasks (25cm^2^). Chromosomes were obtained using colcemid to arrest mitoses, hypotonic treatment with 0.075 M KCl (10 minutes at 37°C), followed by washing in Carnoy fixative (methanol/acetic acid 3:1).

### Classical cytogenetics and Fluorescence in situ hybridization (FISH)

Conventionally stained metaphases (Giemsa 5% in 0.07 M phosphate buffer, pH 6.8) were used for diploid number definition and karyotype ordering. We analyzed slides using the 100x immersion objective of a Leica DM1000 microscope. For each individual, 30 conventionally stained metaphases were captured and analyzed using the *GenAsis* software.

FISH experiments were conducted following standard protocols as described by de Oliveira et al., 2010 [22]. Whole chromosome probes from chicken (GGA), corresponding to pairs 1 to 10 and *Leucopternis albicollis* (LAL) homologous to GGA1 (LAL 3, 6, 7, 15 and 18), 2 (LAL 2, 4, and 20), 3 (LAL 9, 13, 17 and 26), 4 (LAL 1 and 16), 5 (LAL 5), 6 (LAL 3), 7 (LAL 8), 8 (LAL 10), 9 (LAL 12) and 10 (LAL 19) [22], were obtained by flow sorting, and labeled by DOP-PCR with biotin (detected with avidin-CY3) or fluorescein. Chromosomes were counterstained with DAPI. Images were recorded using a 63x immersion objective of a Zeiss Imager2 fluorescent microscope and analyzed with Axionvision 4.8 software (Zeiss, Germany). Comparisons were based on the avian putative ancestral karyotype (PAK), in which pairs PAK 1–11 corresponded to GGA1-GGA3, GGA4q, GGA5-GGA9, GGA4p and GGA10, respectively [23].

## Results

### Karyotype description

**Fig 1:**
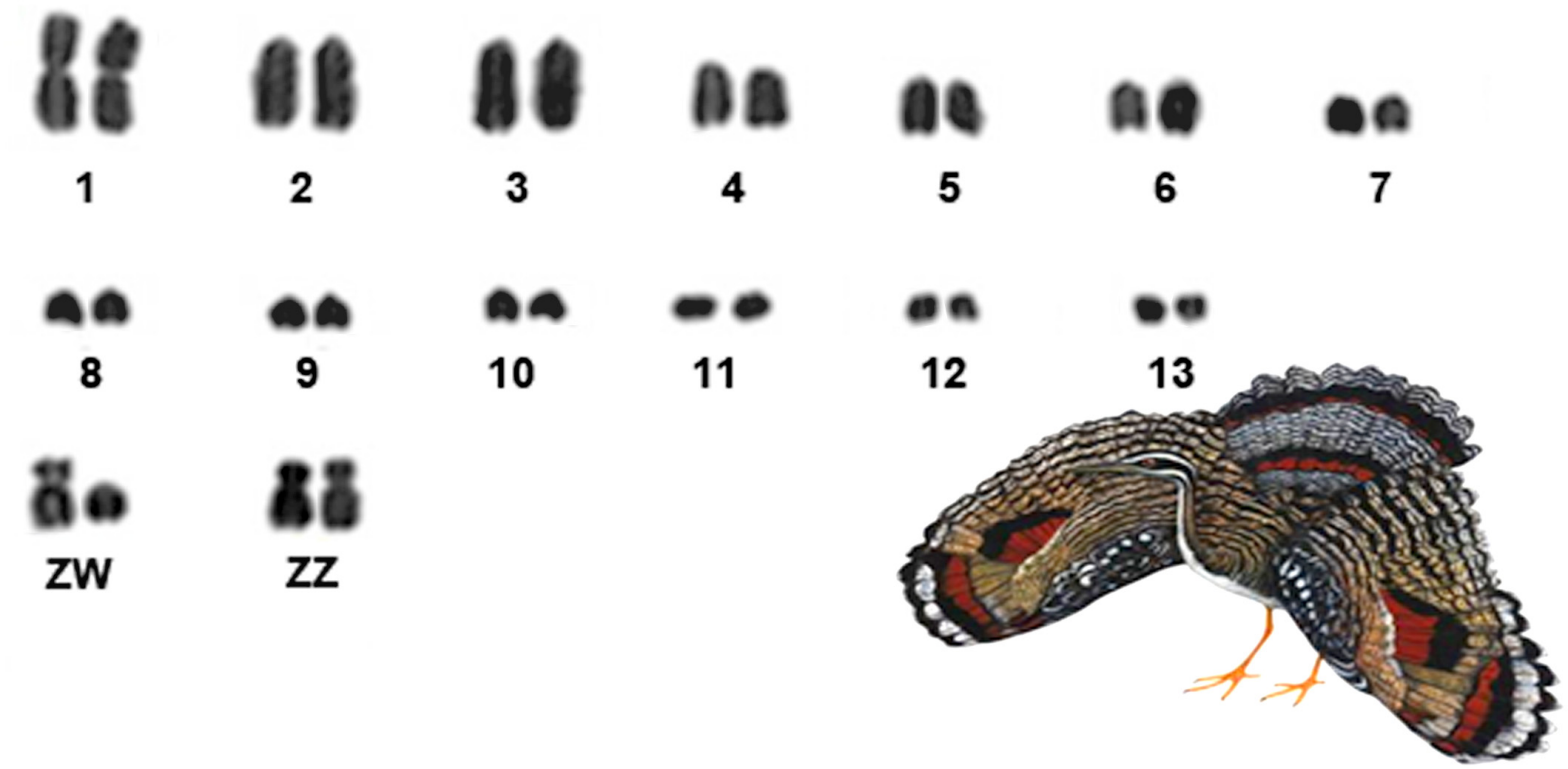
Partial karyotype of sunbittern (*Eurypyga helias)*, 2n = 80, in conventional staining.

The karyotype of *Eurypyga helias* revealed a diploid number of 80, with 10 pairs of macrochromosomes, of which only pair 1 is biarmed, all others being acrocentric (2–10) (Fig 1). The Z chromosome is submetacentric, with a size between the third and fourth autosomal pairs, and the W is acrocentric, corresponding in size to the sixth autosomal pair.

#### Comparative chromosome painting

**Fig 2:**
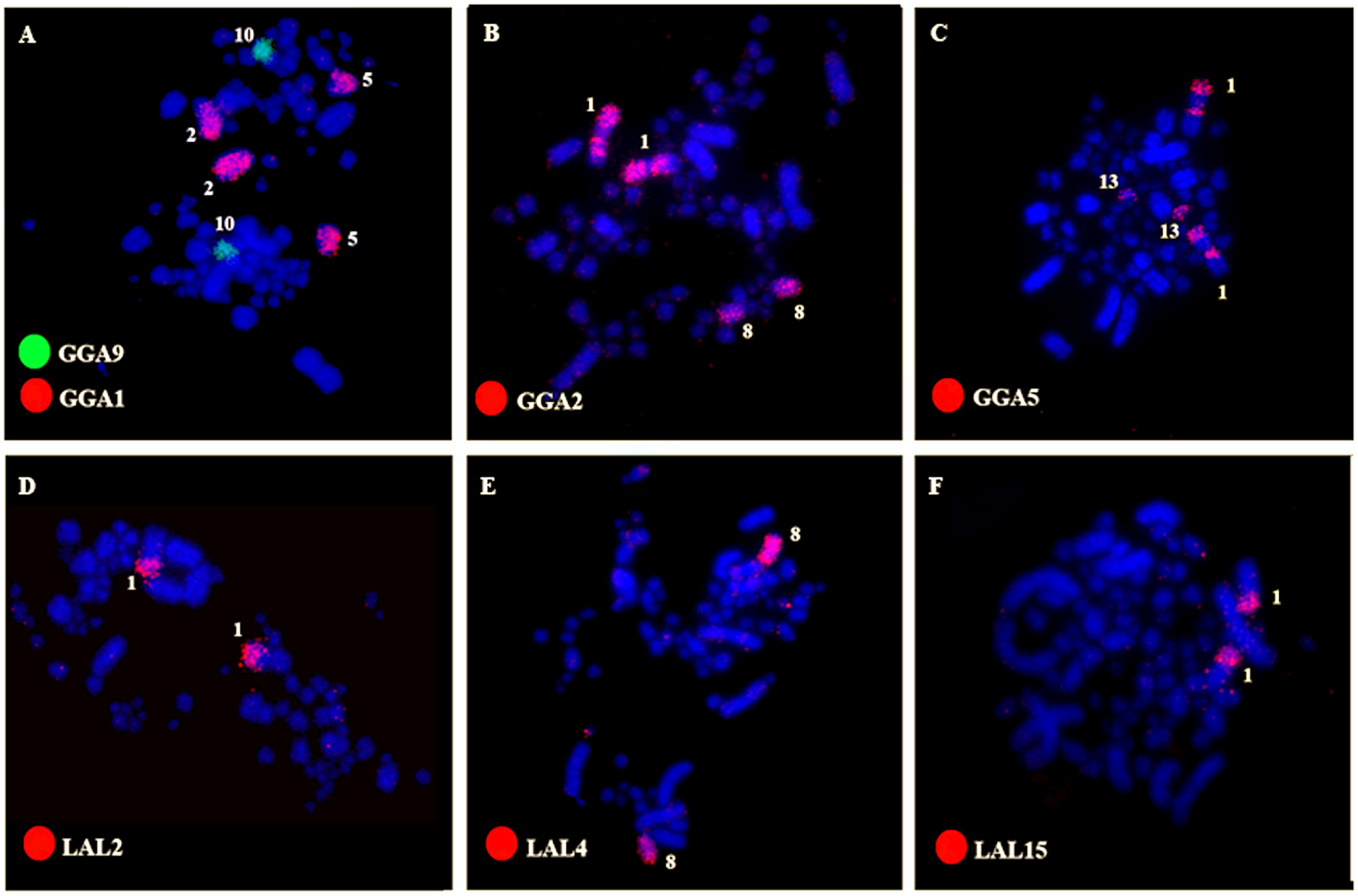
Representative FISH experiments with probes of *G*. *gallus* (A-C) and *L*.*albicollis* (D-F) in metaphases of *Eurypyga helias*, labeled by biotin (red) or fluorescein (green). Chromosome probes are indicated in the left bottom of each figure.

**Fig 3:**
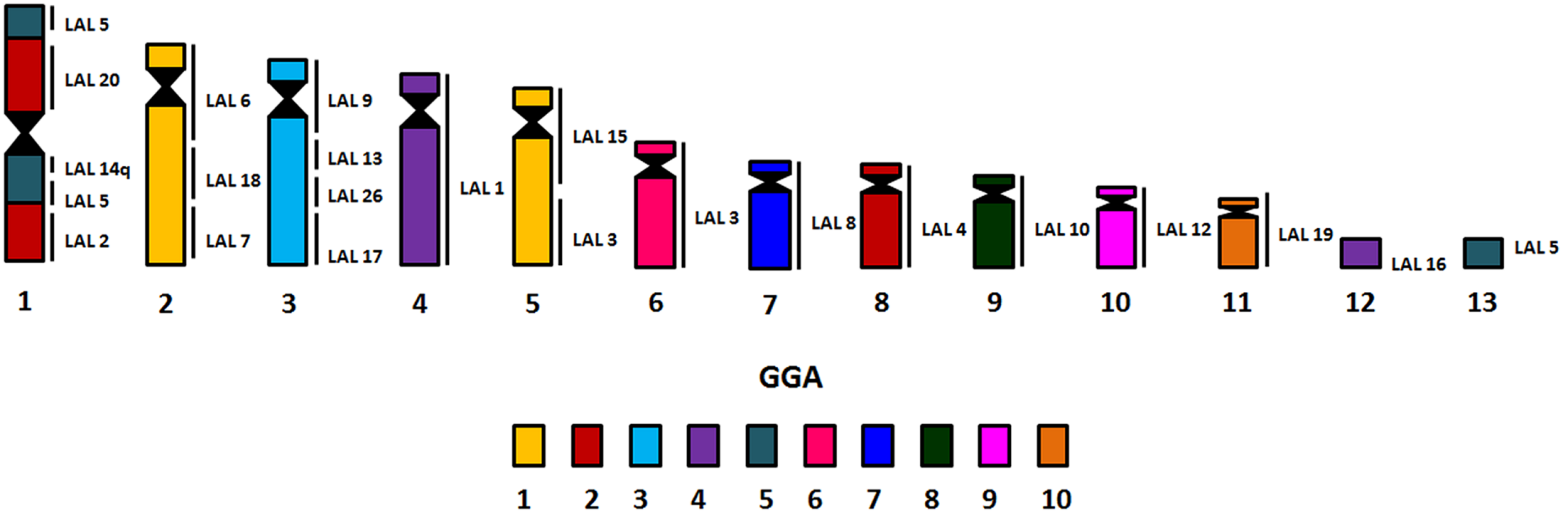
Schematic representation of homologous chromosome segments between *G*. *gallus* and *L*.*albicollis* in *H*. *helias*, detected by *fluorescent in situ* **ab** *hybridization* (FISH).

Chromosome painting with specific probes made from the first 10 autosomal pairs of *G*. *gallus* (GGA) produced 16 signals in *E*. *helias* (EHE). Following the proposed nomenclature of the putative ancestral avian karyotype (PAK), the occurrence of chromosomal fissions was identified in PAK 1, 2 and 5, each of them corresponding to two distinct pairs in EHE (EHE 2 and 5, EHE 1 and 8, EHE 1 and 13, respectively) (Figs 2 and 3). PAK 3, 4, 6, 7, 8, 9 and 10 hybridized to chromosomes EHE 3, 4, 6, 7, 9, 10, and 11, confirming the conservation of autosomal syntenies. In addition, we found a fusion between PAK2q and PAK5q, followed by a pericentric inversion, in EHE1.

**Fig 4:**
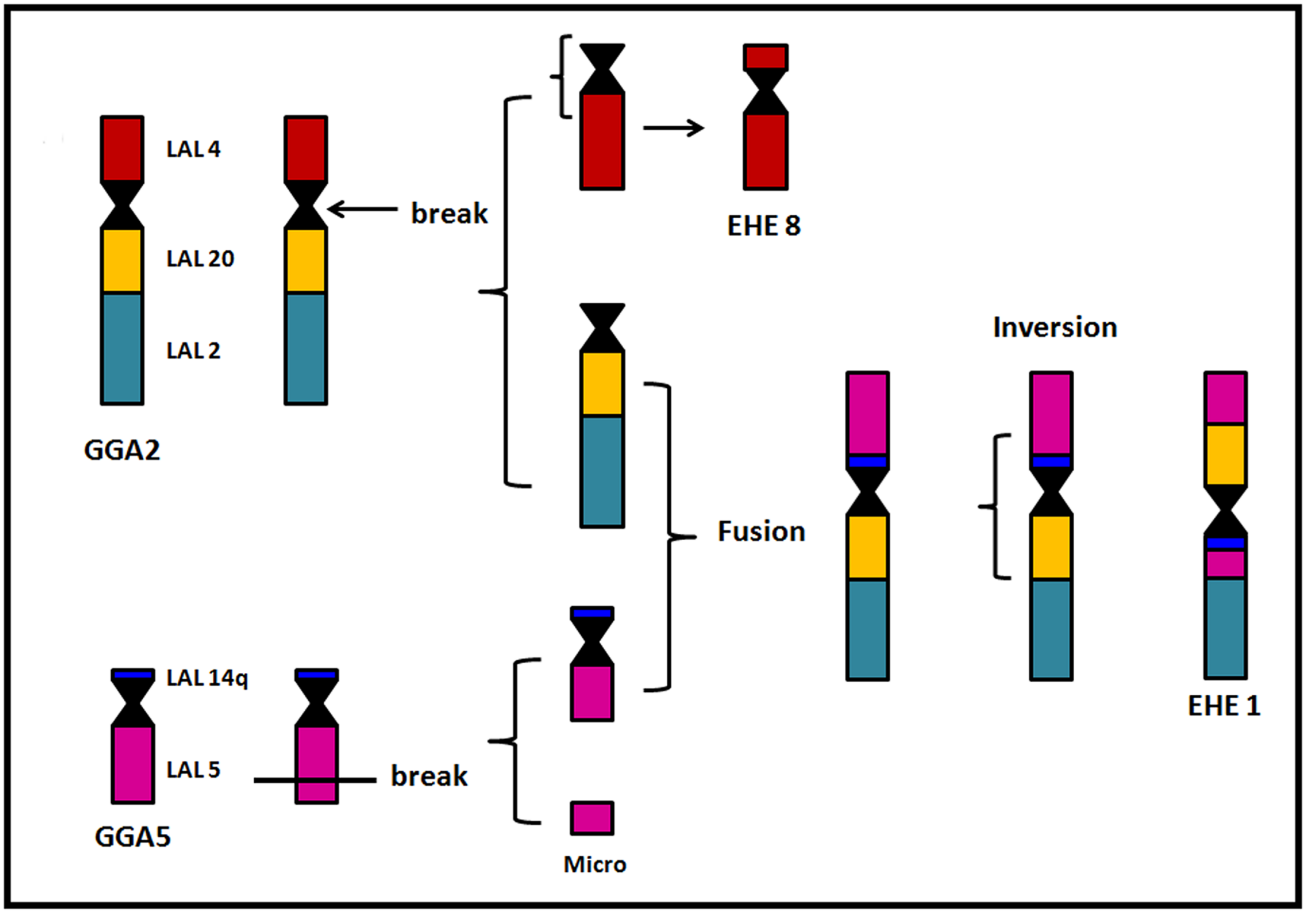
Schematic representation of rearrangements involving pairs EHE1 and 8, based on the results of chromosome painting using probes of *L*.*albicollis* from the regions of GGA 2 and 5.

LAL whole chromosome paints confirmed the results found with *G*. *gallus* probes. Moreover, they allowed the identification of the fission breakpoints in chromosomes corresponding to PAK 1, PAK2 and PAK5. In PAK1, the breakpoint was centric: PAK1q (LAL 6, 18 and 7) corresponded to EHE 2, and PAK 1p (LAL 15 and 3) to EHE 5. The sequence of segments according to these results suggests the occurrence of one pericentric inversion or centromeric shift in EHE 2 and 5. In a similar way, LAL 4 (PAK2p) corresponded to EHE8, indicating that the fission in PAK2 was also at the centromere. In addition, EHE8 underwent a pericentric inversion or centromeric shift. In PAK5, on the other hand, the fission occurred in the long arm (LAL 5), corresponding to EHE 1 and 12 (Fig 4).

## Discussion

### Karyotype description

The sunbitten (*Eurypyga helias*), a gruiform species belonging to the family Eurypygidae, has its karyotype described now for the first time as 2n = 80. This diploid number is similar to that found in most bird species, including other gruiforms. The analysis of other species of this order, including cranes (Gruidae), rails (Rallidae) and the kagu (Rhynochetidae) indicates that their diploid number varies between 72–86 chromosomes [20, 24–30], except for *Cariama cristata* (family Cariamidae) with 2n = 108 [30].

The morphology of the sunbittern macrochromosomes is characterized by the presence of only one metacentric autosomal pair, i.e., chromosome 1, the remainder being acrocentric. This pattern differs from other Gruiformes, which present a high number of biarmed macrochromosomes [20, 24–30] (Table 1). Interestingly, the karyotype of the kagu, described by Wada *et al*. [27] also shows only one pair of biarmed autosomes (the first pair), although there is a slight difference in the length of the short arm when compared to the first pair of the sunbittern. Furthermore, the diploid number of kagu is 2n = 82, with one more pair of microchromosomes than the sunbittern. This karyotype similarity might indicate a close phylogenetic relationship between Eurypygidae and Rhynochetidae, confirming previous molecular, morphologic and biogeographic data [1, 13–14, 31].

**Table 1:** Comparison and morphological classification of macrochromosome pairs of 15 species belonging to five families of Gruiformes. Based on chromosome morphology, these species can be divided in five different groups, indicated by letters A-E (A = 1,2,4–6 biarmed and remaining acrocentric; B = 1–10 biarmed; C = 1,4,7 biarmed and remaining acrocentric,telocentric; D = 1–6 biarmed and remaining telocentric and E = 1 biarmed and remaining acrocentric). Gropus A-D show a higher number of biarmed macrochromosomes, while group E shows a higher number of acrocentric macrochromosomes.

		Morphology of chromosome pairs											
Family	Species	1	2	3	4	5	6	7	8	9	10	References	Groups
Gruidae	*Grus japonensis*	SM	SM	A	SM	SM	M	A	A	A	A	Belterman and Boer, 1984	A
Gruidae	*Grus canadensis*	SM	SM	A	SM	SM	M	A	A	A	A	Belterman and Boer, 1984	A
Gruidae	*Grus Antígone*	SM	SM	A	SM	SM	M	A	A	A	A	Belterman and Boer, 1984	A
Gruidae	*Anthropoides virgo*	SM	SM	A	SM	SM	M	A	A	A	A	Belterman and Boer, 1984	A
Gruidae	*Anthropoides paradisea*	SM	SM	A	SM	SM	M	A	A	A	A	Belterman and Boer, 1984	A
Gruidae	*Bygeranis carunculatus*	SM	SM	A	SM	SM	M	A	A	A	A	Belterman and Boer, 1984	A
Gruidae	*Balearica pavonina*	SM	SM	A	SM	SM	M	A	A	A	A	Kaewmad et al., 2013	B
Psophiidae	*Psophia viridis*	M	M	M	SM	M	M	M	M	M	M	Sasaki and Takagi, 1981	C
Psophiidae	*Psophia crepitans*	SM	A	T	M	A	A	M	A	A	A	Sasaki and Takagi, 1981	C
Psophiidae	*Psophia leucoptera*	SM	A	T	M	A	A	M	A	A	A	Sasaki and Takagi, 1981	C
Rallidae	*Fulica atra*	SM	A	T	M	A	A	M	A	A	A	Hammar, 1970	D
Rallidae	*Gallinus chloropus*	M	SM	M	M	SM	M	T	T	T	T	Hammar, 1970	D
Rallidae	*Porzana albicollis*	M	SM	M	M	SM	M	T	T	T	T	Gionannia and Gionannia, 1983	B
Rhynochetidae	*Rhynochetos jubatus*	M	M	SM	SM	SM	M	M	M	M	M	Wada et al., 1993	E
Eurypygidae	*Eurypyga helias*	SM	A	A	A	A	A	A	A	A	**A**	Present work	E

Legend: M, metacentric; A, acrocentric; SM, submetacentric;T, telocentric).

The karyotype similarity between the sunbittern and the kagu, each species confined to different geographic regions remote from one another, can be explained by the hypothesis of Cracraft [1] who suggested that the Gruiformes arose from a common ancestor in Gondwana. The fragmentation of this supercontinent sometime in the Cretaceous isolated the ancestral lineage of the sunbittern in South America, the kagu in New Caledonia and the extinct *Aptornis* in New Zealand [1, 12]. Hence, the similar karyotypes of the sunbittern and kagu arose from a common ancestor living before the Gondwanan vicariance. The timing of sunbittern-kagu differentiation is still under discussion; Fain & Houde [4] suggest that such divergence occurred before the Oligocene, whereas Ericson *et al*. [13] propose it must have occurred during the Oligocene (60 mya). A geological analysis by Ladiges & Cantrill [2] concluded that the land that linked New Caledonia with the continent probably did not become submerged until the early Eocene (55 mya).

### Chromosome painting with *G*. *gallus* and *L*. *albicollis* probes

The number of publications using chromosome-specific probes in birds is still limited. Within the Gruiformes, so far, only two species of the family Rallidae have been studied: the common moorhen (*Gallinula chloropus*), a cosmopolitan bird that occurs also in Brazil, and the coot (*Fulica atra*), found in Europe, Asia and Oceania [20]. Our study of *E*. *helias* using molecular cytogenetics with *G*. *gallus* and *L*. *albicollis* probes provides more detailed information about the karyotype evolution of Gruiformes. Moreover, our results indicate that *E*.*helias* presents chromosomal characteristics that place this species more distantly from other members of the family Rallidae, corroborating results obtained from sequences of nuclear and mitochondrial DNA [9, 13–14].

The probes of *G*. *gallus* correspond to 16 homologous segments in *E*. *helias* rather than the 13 previously found in *Fulica atra* and *Gallinula chloropus* [20]. This difference is due to the fission of PAK 1 and 2 found in the sunbittern, and not identified previously in Rallidae species (Table 2). Another autapomorphy found in EHE is the fusion between PAK2q and PAK5q, followed by an inversion. Based on classical cytogenetics, it is suggested that the fissions of PAK1 and PAK2 are also present in the kagu [27]. Apart from the latter, these fission events were not previously described in Gruiformes, although it is known that the majority of the karyotype changes in birds are related to these two syntenic groups [23]. The probes of *L*. *albicollis* indicated that the sunbittern maintained the same order of segments observed in in *Gallus gallus* and *Cathartes aura* [32] (i.e. LAL 15 and 3 in GGA1p, and LAL 16, 18 and 7 in GGAq). Hence, although pericentric and paracentric inversions involving PAK1 have been described in other orders of birds, such as in Passeriformes [33–34], they were not detected in the sunbittern.

**Table 2:** Correspondence between syntenic groups of Gruiformes species—*Gallinula chloropus* (GCH), *Fulica atra* (FAT), and *Eurypyga helias* (EHE)—and *Gallus gallus* (GGA) and the putative ancestral avian karyotype (PAK): *Gallinula chloropus* (GCH), according to Nanda *et al*. [20], Griffin *et al*. [23], and this study

**Taxa**	**Chromosome pairs**										
GGA	1	2	3	4q	5	6	7	8	9	4p	10
PAK	1	2	3	4	5	6	7	8	9	10	11
EHE	2/5	1/8	3	4	1/13	6	7	9	10	12	11
GCH	1	2	3	4p	4q/12	5q	5p	6	8	7/13	9
FAT	1	2	3	4p	4q/12	5q	5p	6	8	7/13	9

The syntenic group corresponding to EHE1 is the first example of fusion between PAK2q/PAK5q in birds, as well as the inversion involving these segments, which correspond to two autapomorphies, if we consider each rearrangement. The most likely sequence of events would be the centric fission in PAK2 and a fission in PAK5q, followed by the fusion of PAK2q/PAK5, and a subsequent inversion (Fig 4). On the other hand, the fusion of PAK 6 and 7, observed in members of Rallidae [20], was not detected in the sunbittern, in which these probes hybridized onto one pair each. The remaining sunbittern chromosome pairs show conservation of the syntenies found in the putative avian ancestral karyotype.

### Phylogenetic analysis

The taxonomic classification of Gruiformes has been the subject of discussion for more than a century [10], and many different proposals were put forward aimed at elucidating the evolutionary relationships among the families that compose this order. Analysis based on mitochondrial and nuclear DNA proposed a monophyly for five families: Aramidae, Gruidae, Psophidae, Helionithidae and Rallidae (named as “core Gruiformes”). However, Eurypygidae and Rhynochetidae were thought to be more distant to other families and to have a common ancestor. Moreover, studies based on DNA sequencing have placed Cariamidae near falcons [14, 35], a proposal that has been supported by cytotaxonomy, as *Cariama cristata* shows a 2n = 108, with only acrocentric/telocentric elements [30], similar to two species of Falconidae, *Polyborus plancus* and *Milvago chimachima*, each with 2n = 98 and only acrocentric/telocentric elements [30, 36].

**Fig 5:**
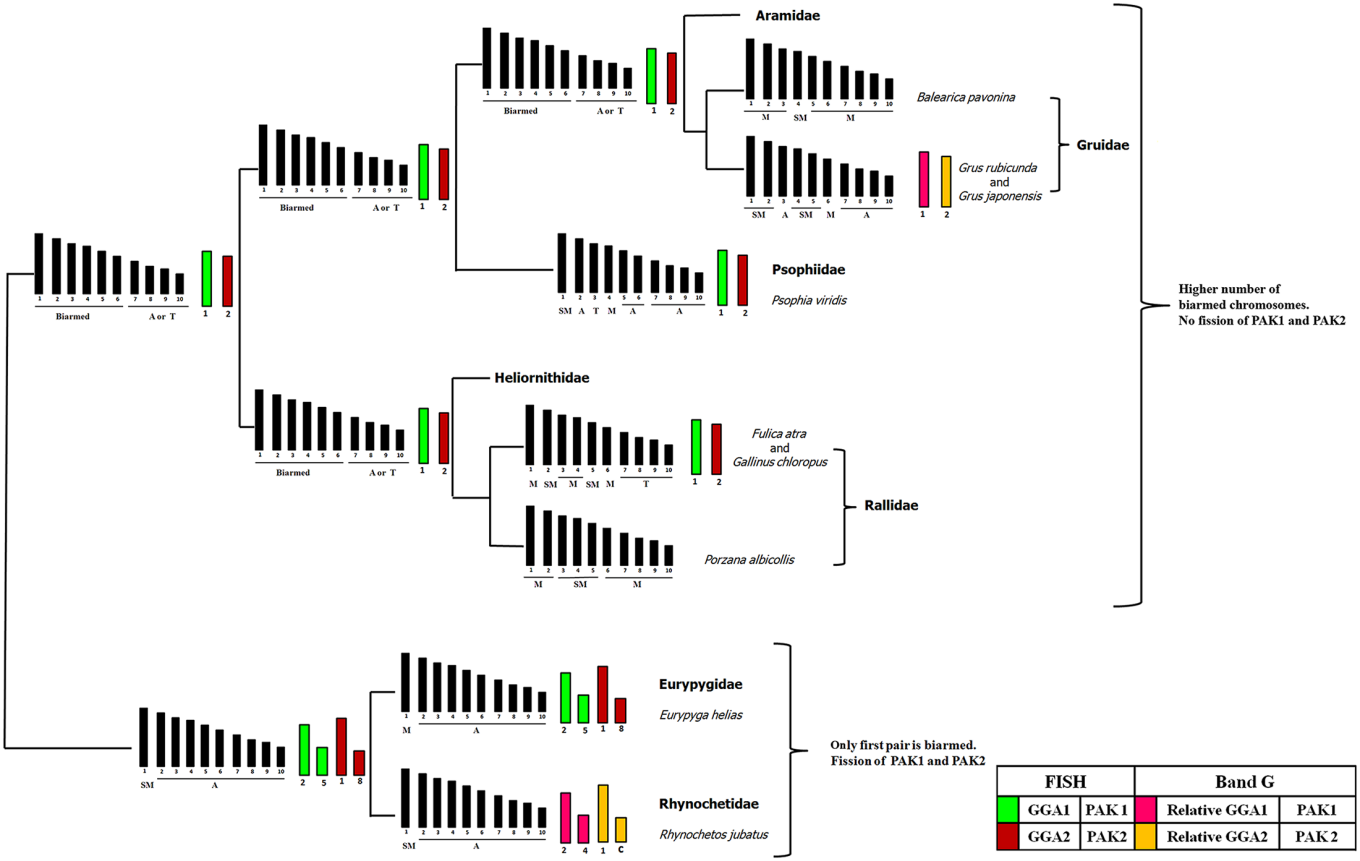
Schematic representation of chromosome rearrangements during the evolution of the Gruiformes based on conventional staining and FISH results [21, 23, 25–27, 37] using a molecular phylogeny constructed from the sequencing of nuclear genes [15]. Data concerning *Psophia viridis* (Psophiidae) were based on unpublished results. We propose that the fissions in PAK 1 and 2 were found in the common ancestor of *Eurypyga helias* and *Rhynochetos jubatus*, but not in the "core Gruiformes" ancestor. (Legend: M, metacentric; A, acrocentric; SM, submetacentric;T, telocentric; PAK, putative ancestral avian karyotype; c, pair not described).

The karyotypic analysis obtained by chromosome painting with *G*. *gallus* and *L*. *albicollis* probes, that shows especially the ancestral chromosome GGA4 (PAK 4 and 10), GGA1 (PAK 1) and GGA2 (PAK 2), supports the distant position of Eurypygidae (*Eurypyga helias*) in relation to core Gruiformes (Fig 5). In the putative ancestral avian karyotype, chromosome 4 probably corresponds to two pairs, as in the majority of birds [17, 23, 32–34, 37]. So far, in Gruiformes, it is possible to identify three different states of this synteny: (1) corresponding to two pairs, such as in the ancestral karyotype (Family Eurypygidae); (2) corresponding to three pairs due to the fission in PAK10 (GGA4p) (Family Psophidae); and (3) the fission of GGA4 and also the fusion GGA4/GGA5 (Rallidae) [20]. These particularities are consistent with the hypothesis that places the Eurypygidae in an external position in relation to the core Gruiformes, since the sunbittern has GGA 4 (PAK 4 and 10) corresponding to the first state (two chromosomes pairs, a plesiomorphic characteristic) and, on the other hand, has several fissions not shared with Rallidae, as in PAK1 and PAK2.

In addition to the phylogenetic proposals based on nuclear DNA sequence, the results obtained from chromosome painting support a hypothesis of two distinct ancestral karyotypes for the seven families that comprise this group. The Eurypygidae and Rhynochetidae, seem to form a monophyletic group in which their common ancestor has fissions of PAK1 and PAK 2. On the other hand, the putative common ancestral karyotype of the core Gruiformes is proposed to have a second fission on PAK10, although this needs to be confirmed in other species of this order. However, PAK1 and PAK2 should be conserved in this karyotype. Although it is essential to have molecular cytogenetic studies in additional Gruiformes species, the results obtained so far suggest that the sunbittern is not included in the monopyletic group and reinforce its relationship with the kagu.
