# Correction: Cytotaxonomy of *Eurypyga helias* (Gruiformes, Eurypygidae): First Karyotypic Description and Phylogenetic Proximity with Rynochetidae

**DOI:** 10.1371/journal.pone.0147458

**Published:** 2016-01-14

**Authors:** Ivanete de Oliveira Furo, Amanda Almeida Monte, Michelly da Silva dos Santos, Marcella Mergulhão Tagliarini, Patricia C. M. O´Brien, Malcolm A. Ferguson-Smith, Edivaldo H. C. de Oliveira

A row has been deleted from Table 1 in the published article. Please see the complete, correct, Table 1 here.

**Table 1:** Comparison and morphological classification of macrochromosome pairs of 15 species belonging to five families of Gruiformes. Based on chromosome morphology, these species can be divided in five different groups, indicated by letters A-E (A = 1,2,4–6 biarmed and remaining acrocentric; B = 1–10 biarmed; C = 1,4,7 biarmed and remaining acrocentric,telocentric; D = 1–6 biarmed and remaining telocentric and E = 1 biarmed and remaining acrocentric). Gropus A-D show a higher number of biarmed macrochromosomes, while group E shows a higher number of acrocentric macrochromosomes.

		Morphology of chromosome pairs											
Family	Species	1	2	3	4	5	6	7	8	9	10	References	Groups
Gruidae	*Grus japonensis*	SM	SM	A	SM	SM	M	A	A	A	A	Belterman and Boer, 1984	A
Gruidae	*Grus canadensis*	SM	SM	A	SM	SM	M	A	A	A	A	Belterman and Boer, 1984	A
Gruidae	*Grus Antígone*	SM	SM	A	SM	SM	M	A	A	A	A	Belterman and Boer, 1984	A
Gruidae	*Anthropoides virgo*	SM	SM	A	SM	SM	M	A	A	A	A	Belterman and Boer, 1984	A
Gruidae	*Anthropoides paradisea*	SM	SM	A	SM	SM	M	A	A	A	A	Belterman and Boer, 1984	A
Gruidae	*Bygeranis carunculatus*	SM	SM	A	SM	SM	M	A	A	A	A	Belterman and Boer, 1984	A
Gruidae	*Balearica pavonina*	M	M	M	SM	M	M	M	M	M	M	Kaewmad et al., 2013	B
Psophiidae	*Psophia viridis*	SM	A	T	M	A	A	M	A	A	A	Sasaki and Takagi, 1981	C
Psophiidae	*Psophia crepitans*	SM	A	T	M	A	A	M	A	A	A	Sasaki and Takagi, 1981	C
Psophiidae	*Psophia leucoptera*	SM	A	T	M	A	A	M	A	A	A	Sasaki and Takagi, 1981	C
Rallidae	*Fulica atra*	M	SM	M	M	SM	M	T	T	T	T	Hammar, 1970	D
Rallidae	*Gallinus chloropus*	M	SM	M	M	SM	M	T	T	T	T	Hammar, 1970	D
Rallidae	*Porzana albicollis*	M	M	SM	SM	SM	M	M	M	M	M	Gionannia and Gionannia, 1983	B
Rhynochetidae	*Rhynochetos jubatus*	SM	A	A	A	A	A	A	A	A	**A**	Wada et al., 1993	E
Eurypygidae	*Eurypyga helias*	M	A	A	A	A	A	A	A	A	**A**	Present work	E

Legend: M, metacentric; A, acrocentric; SM, submetacentric;T, telocentric).
